# Characterization of the Role of Amylo-Alpha-1,6-Glucosidase Protein in the Infectivity of *Toxoplasma gondii*

**DOI:** 10.3389/fcimb.2019.00418

**Published:** 2019-12-06

**Authors:** Xue-Zhen Cao, Jin-Lei Wang, Hany M. Elsheikha, Ting-Ting Li, Li-Xiu Sun, Qin-Li Liang, Zhi-Wei Zhang, Rui-Qing Lin

**Affiliations:** ^1^College of Veterinary Medicine, South China Agricultural University, Guangzhou, China; ^2^Key Laboratory of Veterinary Parasitology of Gansu Province, State Key Laboratory of Veterinary Etiological Biology, Lanzhou Veterinary Research Institute, Chinese Academy of Agricultural Sciences, Lanzhou, China; ^3^Key Laboratory of Zoonosis Prevention and Control of Guangdong Province, Guangzhou, China; ^4^Faculty of Medicine and Health Sciences, School of Veterinary Medicine and Science, University of Nottingham, Sutton Bonington Campus, Loughborough, United Kingdom

**Keywords:** *Toxoplasma gondii*, CRISPR/Cas9, Aa16GL, virulence, brain cyst burden

## Abstract

In this study, we characterized the role of amylo-alpha-1,6-glucosidase (Aa16GL) in the biology and infectivity of *Toxoplasma gondii*, using Aa16GL-deficient parasites of type I RH and type II Prugniaud (Pru) strains. The subcellular localization of Aa16GL protein was characterized by tagging a 3 × HA to the 3′ end of the Aa16GL gene endogenous locus. Immunostaining of the expressed Aa16GL protein revealed that it is located in several small cytoplasmic puncta. Functional characterization of ΔAa16GL mutants using plaque assay, egress assay and intracellular replication assay showed that parasites lacking Aa16GL exhibit a slight reduction in the growth rate, but remained virulent to mice. Although PruΔAa16GL tachyzoites retained the ability to differentiate into bradyzoites *in vitro*, they exhibited slight reduction in their ability to form cysts in mice. These findings reveal new properties of Aa16GL and suggest that while it does not have a substantial role in mediating *T. gondii* infectivity, this protein can influence the formation of parasite cysts in mice.

## Introduction

*Toxoplasma gondii* can infect almost all warm-blooded animals and humans, with reports estimating that nearly one third of world population are chronically infected (Kim and Weiss, 2004; Robert-Gangneux and Dardé, 2012; Wang et al., 2019). *T. gondii* tachyzoites have the ability to invade and replicate inside any nucleated mammalian cells (Hakimi et al., 2017). This parasite must salvage carbon sources and other essential nutrients (e.g., nucleotides, proteins, lipids) from their surrogate host cells in order to meet its metabolic needs during growth and proliferation (Jacot et al., 2016). *T. gondii* genome encodes a number of glycogenes that can contribute to the assembly of glycophosphatidylinositol (GPI)^6^-anchors, *O*-glycans, *N*-glycans, *C*-mannose, glycolipids, and polysaccharides (Perez-Cervera et al., 2011; Gas-Pascual et al., 2018). These proteins play roles in central carbon metabolism, such as gluconeogenesis and glycolysis in the cytosol, as well as oxidative phosphorylation and tricarboxylic acid cycle in the mitochondria.

Glucose is the major carbon and energy source for *T. gondii* and its assimilation through glycolytic pathway contributes to optimal growth of the parasite (Blume et al., 2009; Rahman et al., 2017; Shukla et al., 2018; Beraki et al., 2019). Although glucose is an important nutrient for *T. gondii*, tachyzoites lacking glucose transporter (Δ*gt1*) or the first glycolytic enzyme, hexokinase (Δ*hk*), remain viable (Blume et al., 2009; Shukla et al., 2018). However, tachyzoites can no longer catabolize glucose salvaged from host through glycolytic pathway in the absence of GT1 or HK protein, and glutamine was found to be essential for the growth and motility of these mutant parasites, indicating that glutamine serves as an alternative nutrient to sustain the carbon and energy requirements in *T. gondii* (Blume et al., 2009; Shukla et al., 2018; Xia et al., 2019). Intracellular *T. gondii* catabolizes host glucose through oxidative tricarboxylic acid (TCA) cycle to generate energy (Seeber et al., 2008). It also catabolizes glutamine through TCA cycle and γ-aminobutyric acid (GABA) shunt, to produce GABA and additional macromolecules that enter the TCA cycle to generate energy (Macrae et al., 2012).

*Toxoplasma gondii* tachyzoites produce polysaccharide amylopectin, which is composed of a backbone of alpha (1–4)-linked glucose modified with alpha (1–6)-linked branch points (Coppin et al., 2003; Guérardel et al., 2005). *T. gondii* tachyzoites usually generate a low level of amylopectin unless stressed. However, oocysts and bradyzoites accumulate a high level of amylopectin granules in their cytoplasm (Ferguson et al., 1974; Dubey et al., 1998; Rougier et al., 2017). Amylopectin granules serve as an energy reserve during parasite transmission to sustain the parasite's viability in low-nutrient niches and/or to promote rapid differentiation when conditions become favorable (Coppin et al., 2003; Guérardel et al., 2005). Ca^2+^-dependent protein kinase (CDPK2) plays an important role in the regulation of amylopectin formation and degradation and its deletion causes excessive accumulation of amylopectin and death of the parasite cysts in mice (Uboldi et al., 2015). CDPK2 can also phosphorylate starch-metabolic enzymes, such as glycogen phosphorylase (GP), pyruvate phosphate dikinase, alpha-glucan water dikinase and amylo-alpha-1,6-glucosidase (Aa16GL) (Uboldi et al., 2015). Glycogen phosphorylase plays a role in the regulation of starch digestion and its loss can also cause accumulation of starch and reduction of parasites cysts in mice (Silver et al., 2014; Mahlow et al., 2016; Sugi et al., 2017). Although Aa16GL is a major enzyme for degradation of glycogen in the human body (Arad et al., 2005), its role in *T. gondii* infectivity is unclear (Uboldi et al., 2015).

Here, we used CRISPR-Cas9 gene editing technology to study the subcellular localization and biological roles of Aa16GL in *T. gondii* infectivity. Our results showed that Aa16GL was localized predominantly to several small puncta within the cytoplasm. Deletion of Aa16GL did not significantly reduce parasite replication, egress and virulence in mice or the regulation of starch digestion, however cyst-forming ability was reduced in mice.

## Materials and Methods

### Mice and Parasite Strains

Female, 8-week-old, C57BL/6 mice were purchased from Lanzhou University Laboratory Animal Center, Lanzhou, China. During the experiment, all mice (10 mice/group) were raised in SPF environment of animal care facilities. Tachyzoites of *T. gondii* type I (RH strain) and type II (Pru strain) were maintained in human foreskin fibroblast (HFF) cell (HFF, ATCC, Manassas, VA, USA) monolayers in Dulbecco's modified Eagle's medium (DMEM) supplemented with 2% fetal bovine serum (FBS), 10 mM HEPES (pH 7.2), 100 U/ml penicillin and 100 Ug/ml streptomycin at 37°C with 5% CO_2_, as previously described (Bai et al., 2018).

### Construction of Aa16GL Knockout Strains by CRISPR-Cas9 System

CRISPR-Cas9 system was used to disrupt Aa16GL gene as previously described (Shen et al., 2014; Wang et al., 2016). Briefly, Aa16GL-specific CRISPR-Cas9 plasmid was constructed by replacing the UPRT targeting guide RNA (gRNA) in pSAG1-Cas9-sgUPRT with corresponding gRNAs, using Q5 site-directed mutagenesis, as previously described (Wang et al., 2016). To prepare the homologous templates, the 5′- and 3′-homologous arms of Aa16GL were amplified in the DNA of RH strain, and the DHFR sequences were amplified from the plasmid pUPRT-DHFR-D. Then, these fragments were cloned into pUC19 plasmids by multi-fragment cloning using the ClonExpress II one-step cloning kit (Vazyme Biotech Co., Ltd, Nanjing, China) to generate 5HR-DHFR-3HR, and the positive plasmid was confirmed by DNA sequencing. Approximately 40 μg of CRISPR-Cas9 plasmids and 10 μg of 5HR-DHFR-3HR fragments were mixed and co-transfected into *T. gondii* RH and Pru strains. Single stable clones were screened with 3 μM pyrimethamine and using limiting dilution in 96-well plates (Shen and Sibley, 2014; Wang et al., 2016). Diagnostic PCRs and RT-PCR were used to check the disruption of Aa16GL gene. RT-PCR were performed as previously described (Bai et al., 2018). All primers used in this study are listed in Table S1.

### C-Terminal Tagging

C-terminal endogenous tagging was performed as described previously (Liu et al., 2019). Briefly, a homology region of about 1.5 kb covering the 3′ region of Aa16GL gene, without the STOP codon, was amplified by PCR using genomic DNA of RH strain as a template. The PCR product was inserted into p3HA-LIC-DHFR plasmids. The constructs were linearized and co-transfected with a C-terminal Aa16GL-specific CRISPR-Cas9 plasmids into RH strain. Positive clones were selected by PCR using primers located in the C-terminal Aa16GL-specific CRISPR-Cas9 targeted region, followed by Western blotting and immunofluorescence analysis.

### Western Blotting Analysis and Immunofluorescence Assay

Rabbit anti-HA monoclonal antibody (MAb) and mouse anti-HA MAb purchased from Cell Signaling Technology, mouse anti-SAG1 MAb, mouse anti-GRA5 MAb, polyclonal rabbit anti-MIC2, and polyclonal rabbit anti-aldolase prepared in our laboratory were used in this study. For Western blotting analysis, the purified tachyzoites were lysed on ice with RIPA buffer to harvest the proteins, and then the proteins were subjected to SDS-PAGE analysis, followed by transfer to polyvinylidene fluoride (PVDF) membrane (Immobilon, Millipore) by wet electroblotting as previously described (Anderson-White et al., 2011).

For immunofluorescence analysis, tachyzoite-infected HFFs were fixed with 4% paraformaldehyde. After permeabilization with 0.2% Triton X-100, cells were blocked with 5% bovine serum albumin (BSA) and incubated with mouse anti-SAG1 (1:1,000) MAb, mouse anti-GRA5 (1:500) MAb, rabbit anti-MIC2 (1:500) Ab and rabbit or mouse anti-HA (1:1,000) MAb diluted with 1% BSA for overnight. After washing 5 times with PBS, cell preparations were incubated at 37°C for 1 h with Alexa Fluor 594 goat anti-mouse IgG (H+L) (1:1,000) and Alexa Fluor 488 goat anti-rabbit IgG (H+L) (1:1,000) (Invitrogen, USA). Cells were imaged by a Leica confocal microscope system (TCS SP52, Leica, Germany) as previously described (Wang et al., 2018).

### Assessment of Parasite Growth Kinetics Using Plaque Assay

Plaque assay was performed as previously described (Wang et al., 2016). Briefly, tachyzoites were added to 12-well tissue culture plastic plates containing confluent monolayers of HFF cells at ~ 100 tachyzoites of RH strain or 300 tachyzoites for Pru strain per well. The culture plates were incubated for 7 or 9 days at 37°C and 5% CO_2_. Then, cells were then fixed with 4% paraformaldehyde and stained with 0.2% crystal violet. The number and size of plaques were analyzed using a scanner.

### Intracellular Replication of *T. gondii*

Intracellular replication assay was performed as previously described (Wang et al., 2016). HFF cells were infected with 10^5^ tachyzoites/well. One hour later, the cells were washed with PBS to remove tachyzoites that remained extracellular and the plates were incubated for further 23 h. The cell samples were then fixed with 4% paraformaldehyde and stained with mouse anti-SAG1, followed by Alexa Fluor 488 goat-anti mouse IgG. The numbers of parasitophorous vacuoles (PVs) containing 1, 2, 4, 8, or 16 parasites were counted. At least 200 PVs were analyzed for each strain. Data are presented as means ± standard deviations (SDs) from three independent experiments.

### Egress Assay

HFF cell monolayers maintained in 12-well culture plates were infected with freshly egressed tachyzoites (tachyzoites per well). After 1 h, the extracellular tachyzoites were washed off with PBS, and then fresh DMEM medium was added and the cultures were incubated for further 30–36 h. Intracellular parasites were then treated with 3 μM calcium ionophore A23187 or DMSO, and time-lapse imaging was performed using live microscopy as previously described (Wang et al., 2016).

### Sensitivity of Extracellular Tachyzoites to Carbon Source Limitation

Tachyzoites were re-suspended in DMEM containing 2% FBS (regular DMEM) or PBS (as carbon source-free medium) in a humidified atmosphere at 37°C and 5% CO_2_ for 6 h, and then the supernatants were removed by centrifugation at 1,000 g for 5 min. The parasites were re-suspended in DMEM containing 2% FBS and counted. Then, plaque formation assay was performed to determination of the sensitivity of extracellular tachyzoites to carbon source-free medium by culturing the treated parasites in monolayers of HFFs, for 9 days at 37°C and 5% CO_2._ The sensitivity rate was calculated by dividing the size of plaque formed by parasite that was treated with DMEM containing 2% FBS by the size of plaque formed by parasite treated with PBS. Six independent experiments were performed.

### Transmission Electron Microscopy

Monolayers of HFFs were infected with wild-type Pru or PruΔAa16GL strain for 20 h and then fixed with 2.5% glutaraldehyde in 0.1 M sodium cacodylate buffer (pH 7.4) for 2 h at ambient temperature. Samples were then post-fixed in 1% osmium tetroxide for 1 h and processed as described previously (Uboldi et al., 2015). Images were acquired with a HITACHI HT7700 electron microscope under 80 kV.

### Mouse Infection With Aa16GL-Deficient Strains

The freshly harvested tachyzoites of Aa16GL-deficient or wild type strain were counted and then intraperitoneally (i.p.) injected into 8-week-old female mice (10 mice/strain, 100 tachyzoites/mouse for RH strain and 5,000 tachyzoites/mouse for Pru strain). Negative control mice were only injected with equal volume of PBS. All the mice were monitored daily for signs of disease.

### Bradyzoite Differentiation

Bradyzoite differentiation were performed as described previously (Yang et al., 2017). Briefly, tachyzoites of Pru and PruΔAa16GL strains were added to a 24-well cell culture plate containing HFFs. One hour later, tachyzoites that remained extracellular were washed with DMEM medium. Differentiation medium [alkaline media (pH 8.2), ambient CO_2_] was added and refreshed every day to maintain the alkalinity of the medium. The samples were then subjected to immunofluorescence analysis. All parasites were stained with goat anti-*T. gondii* antibody and bradyzoites were stained with rabbit anti-TgBAG1 antibody. Samples were imaged with a Leica confocal microscope system (TCS SP52, Leica, Germany).

### Statistical Analysis

Statistical analyses for *in vitro* and *in vivo* experiments were performed using Prism 5 (GraphPad Software Inc., La Jolla, CA, USA) using Student's *t*-tests, or Gehan-Breslow-Wilcoxon test as indicated in the figure legends.

## Results

### Characterization and Localization of Aa16GL

**Figure 1:**
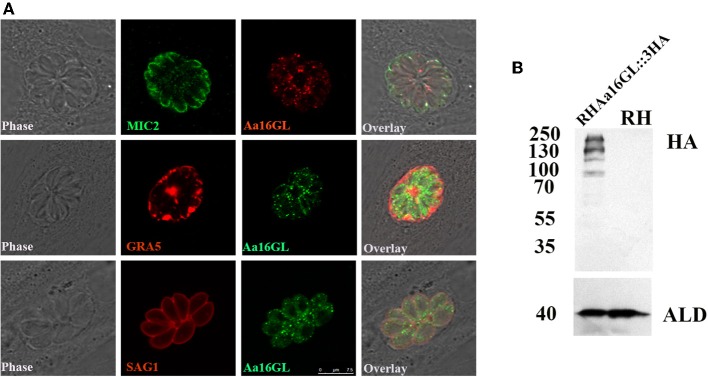
Tagging and localization of amylo-alpha-1,6-glucosidase (Aa16GL). **(A)** Localization of Aa16GL in the cytoplasm with several small puncta by immunofluorescence analysis. **(B)** Western blotting confirmed that 3 × HA tag was successfully inserted into the C-terminal of Aa16GL. The 3 × HA-tagged Aa16GL is about 208 KDa (the highest band), and the other bands may be due to the presence of cleavage or degradation. Anti-aldolase (ALD) served as a loading control.

The Aa16GL gene contained annotated 19 exons (https://toxodb.org) (Gajria et al., 2008). The protein contained 1,882 amino acids with a predicted molecular weight of 208.5 kDa. Aa16GL was expressed in tachyzoites and bradyzoites and the expression levels in both stages were similar. However, its expression level was highest in the unsporulated oocysts (https://toxodb.org). The localization of Aa16GL was investigated by inserting a 3 × HA at the C-terminus of the Aa16GL gene endogenous locus. Successful tagging of the Aa16GL was confirmed by DNA sequencing. IFA results showed that Aa16GL was localized in many small points in the cytoplasm of tachyzoites, similar to the locations of CDPK2 (Figure 1A). Expression of the tagged protein was further verified by Western blotting analysis using anti-HA antibody, which revealed four bands. The size of the largest band was ~208 kDa, which is consistent with the predicted size for Aa16GL protein. The presence of three other bands is perhaps a consequence of internal processing or degradation events. There were no bands detected in the control samples (Figure 1B).

### Disruption of Aa16GL in Type I RH and Type II Pru Strains

**Figure 2:**
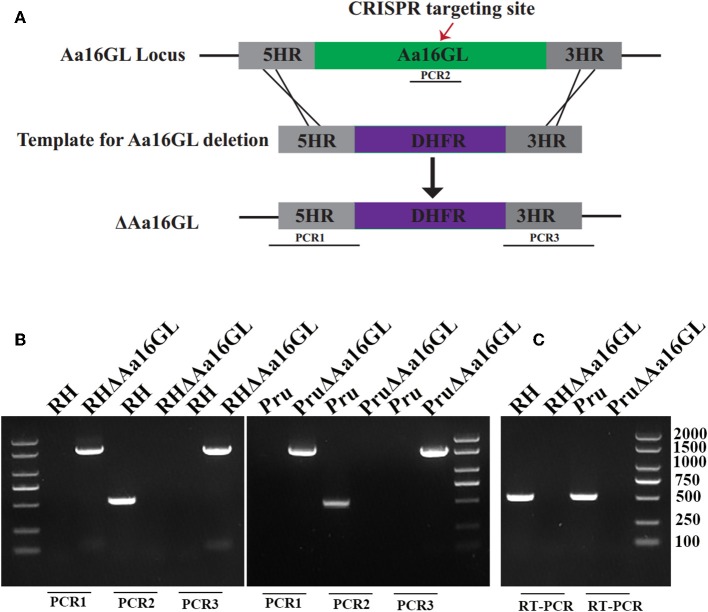
Construction of *Toxoplasma gondii* amylo-alpha-1,6-glucosidase knockout mutant strain. **(A)** Schematic model of deleting Aa16GL by CRISPR-Cas9-mediated homologous gene replacement. **(B)** Diagnostic PCRs show successful knockout of the Aa16GL gene, which was also confirmed at mRNA level by using **(C)** RT-PCR.

To study the biological functions of Aa16GL in the tachyzoite and bradyzoite stages of *T. gondii*, this gene was disrupted in type I RH strain and type II Pru strain by CRISPR-Cas9-mediated homologous recombination technology (Figure 2A). The *Aa16GL* targeting CRISPR-Cas9 plasmid and homology template 5HR-DHFR-3HR were co-transfected into RH or Pru tachyzoites. Single clones were obtained using drug selection and limiting dilution. Diagnostic PCRs and RT-PCR were used to confirm the gene deletion at the DNA and RNA levels, respectively (Figures 2B,C). Our results showed that Aa16GL gene was completely deleted by homologous recombination and that ΔAa16GL mutant was successfully constructed.

### *In vitro* Characterization of the Growth of Aa16GL Mutants

**Figure 3:**
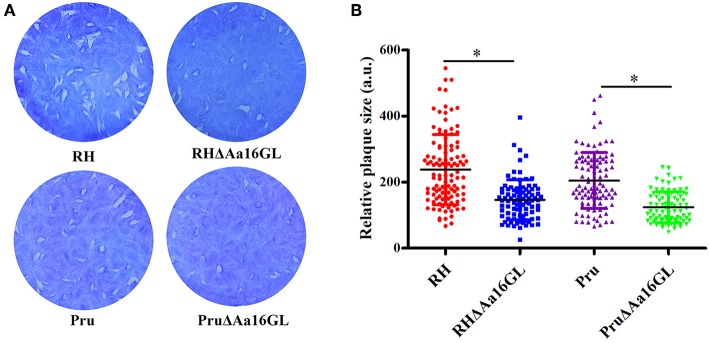
Plaque assay comparing the growth of amylo-alpha-1,6-glucosidase mutant to that of their parental strain. **(A)** Plaque assay of the Aa16GL mutants and their parental strains. **(B)** The sizes of plaques generated by Aa16GL mutants were significant smaller than that generated by their parental strains (^*^P < 0.05). Differences were significant in all three experiments analyzed by Student's *t*-tests.

Plaque assay was performed in order to assess the growth capability of the Aa16GL mutant strain. Aa16GL mutant strains and their parental strains were allowed to grow for several lytic cycles over a period of 7 (for RH and RHΔAa16GL strains) or 9 (for Pru and PruΔAa16GL strains) days in confluent HFF monolayers, and then stained with crystal violet. Results showed that the number and size of plaques produced by RHΔAa16GL and PruΔAa16GL strains were slightly fewer and smaller than that of the parental strains (Figure 3). Consistent with the low fitness scores from a genome wide disrupted survey (Sidik et al., 2016), the contribution of Aa16GL to the infectivity of *T. gondii* tachyzoite does not seem to be substantial.

**Figure 4:**
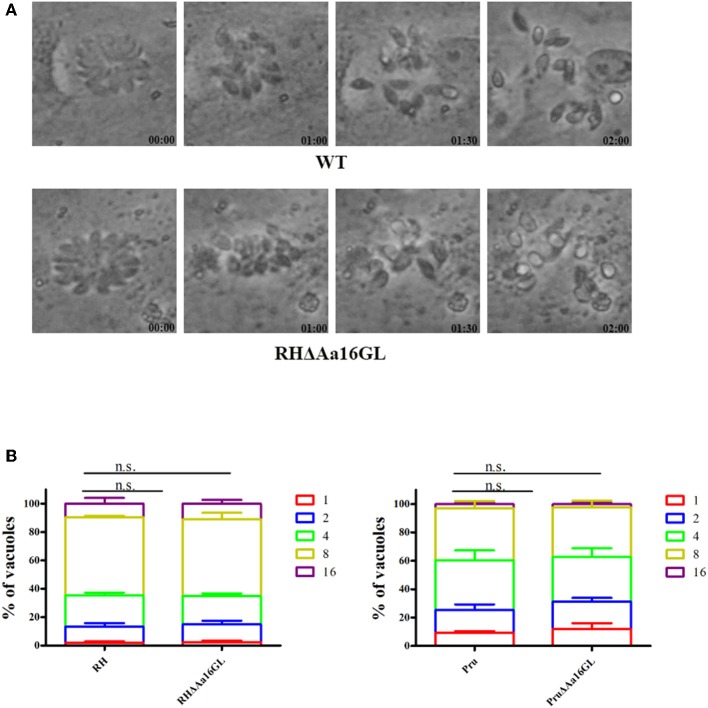
Egress and intracellular replication of amylo-alpha-1,6-glucosidase-deficient tachyzoites *in vitro*. **(A)** The parasite egress of the parental WT (RH strain) and Aa16GL mutant (RH strain). A similar egress pattern was observed between WT strain and Aa16GL mutant strain after addition of 3 μM calcium ionophore A23187 into culture medium. **(B)** WT (RH strain, Pru strain) and corresponding Aa16GL mutant strains were allowed to infect HFF monolayers for 24 h, and the numbers of vacuoles containing 1, 2, 4, 8, or 16 parasites were counted. At least 200 vacuoles were analyzed for each strain, each was performed in three independent experiments. n.s, not significant, Student's *t*-tests.

To further investigate the effect of Aa16GL on the lytic cycle of *T. gondii*, parasite egress and intracellular growth were investigated. To evaluate the parasite's egress efficiency, tachyzoites were allowed to grow on 12-well culture plates until the majority of the PVs contained ≥16 tachyzoites, and then 3 μM calcium ionophore A23187 were added and the parasite egress rate was determined by time-lapse microscopy over 10 min. Both Aa16GL mutant strain and their corresponding parental strain rapidly egressed from HFF cells after 2 min stimulation with A23187 (Figure 4A). To evaluate the influence of Aa16GL on intracellular parasite's replication, HFF cells were infected with Aa16GL mutant strain and their parental strain. Twenty-four hours later, cells were fixed with 4% paraformaldehyde, and then the number of tachyzoites within the PVs was counted. Results showed that the intracellular replication kinetics of Aa16GL mutant strain and the parental strain is comparable (Figure 4B). These results show that the slight reduction in the growth capabilities of Aa16GL mutant was not attributed to the parasite's ability replicate or egress from infected host cells.

### Carbon Source Limitation Does Not Affect Aa16GL Parasite Growth

**Figure 5:**
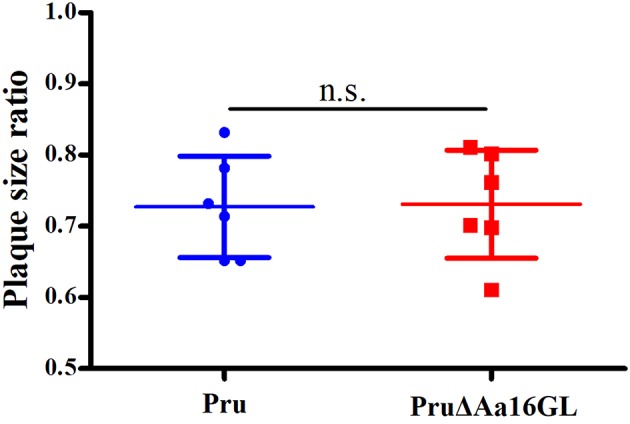
Effect of external carbon sources on *Toxoplasma gondii* growth. Parental Pru strain or PruΔAa16GLstrain treated with regular DMEM or carbon source-free medium for 6 h were allowed to infect HFF monolayers under normal conditions for 9 days. Samples were fixed and the plaque sizes were determined. n.s, not significant, Student's *t*-tests.

Identical numbers of extracellular parasites collected carbon-free medium or DMEM were used to infect HFF monolayers grown in regular DMEM to examine whether mutant tachyzoites are more sensitive to extracellular permanence without an external source of carbon. The sensitivity rate was determined and results as shown in Figure 5 suggest that Aa16GL mutant tachyzoites are not more sensitive to extracellular permanence without an external source of carbon.

### Evaluation of Aa16GL on Amylopectin Digestion

**Figure 6:**
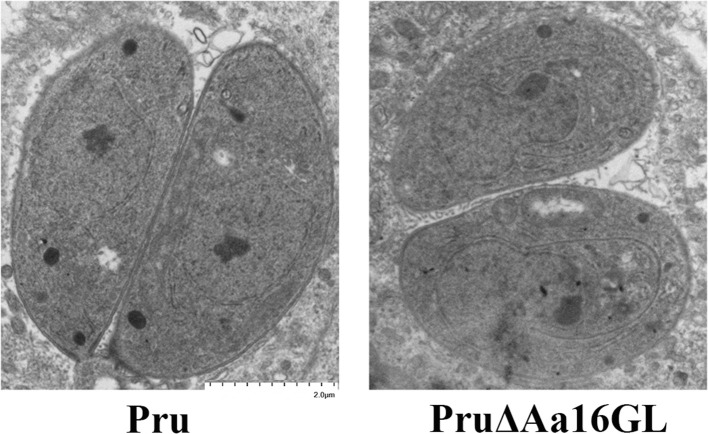
Evaluation of amylopectin accumulation in the Aa16GL mutant tachyzoites by transmission electron microscope. Parental Pru strain or PruΔAa16GL mutant strain was allowed to infect HFF monolayers under normal conditions for 20 h. Samples were fixed and prepared for transmission electron microscope. No amylopectin granules or accumulation of lipid was observed in the PruΔAa16GL mutant or the parental Pru strain.

CDPK2 and GP deficiency can cause aberrant accumulation of amylopectin inside *T. gondii* (Uboldi et al., 2015; Sugi et al., 2017). Aa16GL is an important starch-metabolic enzyme and can be phosphorylated by CDPK2. Therefore, we tested whether deletion of Aa16GL would affect the amylopectin digestion in *T. gondii*. Transmission electron microscopy showed that there was no aberrant amylopectin or lipid accumulation in the tachyzoites of Aa16GL mutants (Figure 6). Also, no obvious amylopectin or lipid accumulation was observed microscopically in the cysts induced *in vitro* by treatment using alkaline media (data not shown).

### Virulence of Aa16GL Mutants in Mice

**Figure 7:**
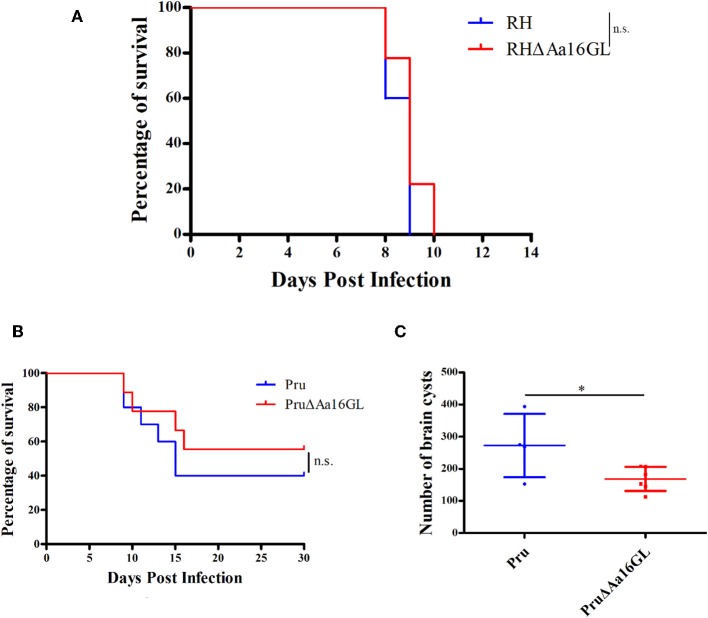
Virulence of amylo-alpha-1,6-glucosidase-deficient strain in mice. **(A)** C57BL/6 female mice (10/group) were intraperitoneally (i.p.) infected with 100 parental RH or RHΔAa16GL tachyzoites, and the survival of mice was monitored until all the mice died (Gehan–Breslow–Wilcoxon tests. n.s. = not significant). **(B)** C57BL/6 female mice (10/group) were i.p. infected with 5 × 10^3^ freshly harvested tachyzoites of Pru or PruΔAa16GL strain. Subsequently their survival was observed for 30 days (Gehan–Breslow–Wilcoxon tests. n.s, not significant, Student's *t*-tests). **(C)** The number of brain cysts in mice was evaluated at day 30 post infection. ^*^P < 0.05, Student's *t*-test.

Results showed that deletion of Aa16GL did not reduce the virulence parasite regardless of the strains used, suggesting that Aa16GL is not a major virulence gene for *T. gondii* in mice (Figures 7A,B). To assess roles of Aa16GL during chronic infection, survived C57BL/6 mice in Figure 7B were sacrificed after 30 days of infection, and the number of brain tissue cysts was determined by microscopic examination. As shown in Figure 7C, the number of brain cysts in mice infected with PruΔAa16GL strain was significantly lower than that detected in the Pru strain (*P* < 0.05). These results indicate that Aa16GL deletion can interfere with *T. gondii* encystation mechanism.

### Bradyzoite Differentiation Assay

**Figure 8:**
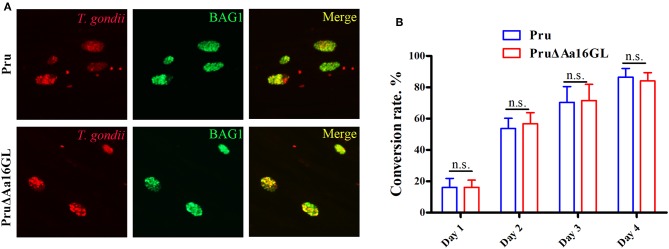
Aa16GL is not necessary for cyst formation *in vitro*. **(A)** Parental Pru strain or PruΔAa16GL mutant strain was allowed to infect HFF monolayers under alkaline conditions (pH 8.2, ambient CO_2_) for 4 days. Cells were fixed and stained with goat anti-*Toxoplasma gondii* antibody (stain of total parasites), and rabbit anti-BAG1 (a bradyzoite specific marker). **(B)** Bradyzoite conversion rates of parental Pru strain or PruΔAa16GL mutant at days 1, 2, 3, and 4 after induction of encystation. n.s, not significant, Student's *t*-tests.

Given that fewer cysts were detected in C57BL/6 mice infected with Aa16GL mutant parasites, we hypothesize that Aa16GL might be involved in the formation of bradyzoites-containing cysts. The ability of Aa16GL mutant to form cysts *in vitro* was examined by inducing tachyzoite-bradyzoite transformation for 1, 2, 3, and 4 days. All parasites labeled with goat anti-*T. gondii* antibody and bradyzoites labeled with rabbit anti-BAG1 were observed by immunofluorescence microscopy. Results showed that *in vitro* differentiation rate of PruΔAa16GL was similar to that of Pru strain (Figures 8A,B).

## Discussion

*Toxoplasma gondii* is an eukaryotic protozoan whose growth is restricted to intracellular environment. This parasite can accumulate amylopectin, which may serve as an energy storage to support its own survival under nutrient-limited conditions (Uboldi et al., 2015). The accumulation of starch-like amylopectin stores has been shown to be a characteristic feature of the cyst stage of *T. gondii* (Coppin et al., 2003; Guérardel et al., 2005; Uboldi et al., 2015). However, the functional significance of amylopectin storage in *T. gondii* remains incompletely understood. CDPK2, a kinase that regulates amylopectin turnover in a Ca^2+^-dependent manner, can phosphorylate starch-metabolic enzymes, such as glycogen phosphorylase and Aa16GA (Uboldi et al., 2015). The functions of glycogen phosphorylase were shown to play role in amylopectin digestion (Sugi et al., 2017). However, the functions of Aa16GA in the context of *T. gondii* infection remains unclear.

In this study, a transgenic parasite that expressed a C-terminally 3 × HA-tagged Aa16GA under their native promoter was constructed to study the cellular localization of Aa16GA. Mutant parasites expressed Aa16GA in their cytosol as several small puncta, similar to what has been observed in CDPK2-deficient *T. gondii* parasites (Uboldi et al., 2015). Furthermore, four bands were observed by Western blotting suggesting that posttranslational modifications may occur, which warrants further investigations.

The biological function of Aa16GA was studied by generating Aa16GL mutants. Consistent with a previous study that genome-wide screening for gene fitness (phenotype enrichment score of −0.24), Aa16GL mutants produced fewer and less-sized plaques than parental strain regardless of the parasite genotype used, indicating that Aa16GL is not an essential gene. The fitness defect was further evaluated by performing egress and intracellular replication assays. However, the egress and replication efficiencies of Aa16GL mutants were similar to their parental strains, suggesting that fitness defect may due to other factors, such as parasite invasion, attachment and survival capacity outside host cells. Finally, the roles of Aa16GL *in vivo* were studied by investigating the survival of mice and the brain tissue cyst's burden in the survived mice after infection of Aa16GL mutants. Again, our results showed that deletion of Aa16GL did not affect the virulence in mice but affect the cyst formation *in vivo*, although loss of Aa16GL did not affect the bradyzoite differentiation. Due to the large size of the Aa16GL protein, we are unable to amplify the full coding sequence of the Aa16GL by RT-PCR, therefore further studies are needed to confirm the roles of Aa16GL in cyst formation in mice by generation of complemented strains.

CDPK2 and GP are also not essential for tachyzoite stage of *T. gondii* survival *in vitro*, however they play important roles in the regulation of amylopectin formation and degradation. Deletion of CDPK2 or GP led to accumulation of amylopectin granules in the tachyzoite basal end and larger granules within the parasite residual body, and this phenotype was further enhanced in type II Pru strain. As Aa16GL is a starch-metabolic enzyme, we evaluated whether deletion of Aa16GL would affect amylopectin accumulation. However, deletion of Aa16GL did not affect amylopectin accumulation even in type II Pru strain using transmission electron microscopy. Because amylopectin granules are also observed in the oocyst stage, further investigation of the roles of Aa16GL in amylopectin metabolism in other life cycle stages is required. On the other hand, carbohydrate metabolism is essential for glycolysis, gluconeogenesis and mitochondrial respiration in *T. gondii* (Al-Anouti et al., 2004; Pomel et al., 2008; Polonais and Soldati-Favre, 2010). Thus, the one could anticipate a serious disturbance of the metabolic pathways associated with the available glucose fluxes. Aa16GL is predicted to be involved in glycolytic fluxes by producing alpha-1,4-glucan and glucose. *T. gondii* tachyzoites are capable of maintaining ATP level without acquisition of external carbon sources during the first hour of its presence extracellularly. However, gliding motility and invasion were affected during long-time deprivation of carbon sources (Lin et al., 2011). Whether Aa16GL mutant tachyzoites are more sensitive to extracellular permanence without an external source of carbon were determined by treating Aa16GL mutant tachyzoites or wild type tachyzoites with PBS or DMEM containing 2% FBS at 37°C for 6 h. Our results showed that Aa16GL mutant tachyzoites are not more sensitive to extracellular permanence without an external source of carbon. These observations can be attributed to the use of internal carbon store by Aa16GL mutant tachyzoites. Further studies are needed to focus on glucose fluxes and other metabolic pathways.

## Conclusions

We have shown that parasites lacking amylo-alpha-1,6-glucosidase (Aa16GL) remain viable, but with slight growth defect. The deletion of Aa16GL did not significantly influence parasites' egress, replication *in vitro* or virulence in mice, but significantly reduced cyst formation in mice. Furthermore, Aa16GL deficiency did not result in unchecked accumulation of starch in tachyzoites. Further studies should investigate whether deletion of Aa16GL affect the glucose fluxes and accumulation of amylopectin in other *T. gondii* life cycle stages, such as oocysts.

## Data Availability Statement

The raw data supporting the conclusions of this manuscript will be made available by the authors, without undue reservation, to any qualified researcher.

## Ethics Statement

The animal study was reviewed and approved by the Animal Administration and Ethics Committee of Lanzhou Veterinary Research Institute, Chinese Academy of Agricultural Science. All experimental mice were handled in strict accordance with the Guidelines and Animal Ethics Procedures of the People's Republic of China.

## Author Contributions

J-LW, R-QL, and HE designed the study and critically revised the manuscript. X-ZC performed the experiments, analyzed the data, and drafted the manuscript. T-TL, L-XS, Q-LL, and Z-WZ participated in the implementation of the study. All authors read and approved the final manuscript.

### Conflict of Interest

The authors declare that the research was conducted in the absence of any commercial or financial relationships that could be construed as a potential conflict of interest.
