# Supplementary material for: Characterization of the Role of Amylo-Alpha-1,6-Glucosidase Protein in the Infectivity of Toxoplasma gondii
Source: Front Cell Infect Microbiol. 2019 Dec 6;9:418. doi: 10.3389/fcimb.2019.00418 (PMC6908810; doi:10.3389/fcimb.2019.00418)
Supplement: Supplementary file 1 [file Table_1.docx]

**TABLE S1｜**Primer used in this study

| Primer | Sequence | Used for |
| --- | --- | --- |
| gRNA-Aa16GL-F | 5′-GGTGGCAGGCCGACGTCGCGGTTTTAGAGCTAGAAATAGC | To construct the special CRISPR line plasmid of Aa16GL |
| gRNA-Aa16GL-R | 5′-AACTTGACATCCCCATTTAC |  |
| U5Aa16GL-Gbison-F | 5′-GGTTTTCCCAGTCACGACGTTACGGTGATAAACCTGAAGATT | Amplification of 5′-homology of Aa16GL for 5H-DHFR*-3H construction |
| U5Aa16GL-Gbison-R | 5′-GGATTTACAGCCTGGCGAAGCTTATGCAGACAGACATCTGTAGT |  |
| U3Aa16GL-Gbison-F | 5′-CTATGCACTTGCAGGATGAATTCGTGCATTTACTTCATTGCGCG | Amplification of 3′-homology of Aa16GL for 5H-DHFR*-3H construction |
| U3Aa16GL-Gbison-R | 5′-GAGCGGATAACAATTTCACAAGAAGCGCAGCGGGGCCGCAG |  |
| U5-In Aa16GL-F | 5′-CCATCTTCCACCTCGCTGACGACTCGTG | PCR1: To verify whether 5'-homology performs accurate homologous recombination |
| U5-In Aa16GL-R | 5′-ATGCAGACAGACATCTGTAGT |  |
| KO-Aa16GL-F | 5′-TCTCCGTCCTGTGCTCTGTCC | PCR2: The knockout of TgAa16GL was verified at the DNA level |
| KO-Aa16GL-R | 5′-GATCGCGTAGTGAACTCCCTTTT |  |
| U3-In Aa16GL-F | 5′-GTGCATTTACTTCATTGCGCG | PCR3: To verify whether 3'-homology performs accurate homologous recombination |
| U3-In Aa16GL-R | 5′-GGTCTCGAGCGCAGGCGTTCTTTCCG |  |
| RT-Aa16GL-F | 5′-TCTCCGTCCTGTGCTCTGTCC | The knockout of TgAa16GL was verified at the RNA level |
| RT-Aa16GL-R | 5′-GATCGCGTAGTGAACTCCCTTTT |  |
| gRNA-3Aa16GL-F | 5′-GGGTGCGCCGGGTGAGATTTGTTTTAGAGCTAGAAATAGC | To construct special CRISPR plasmids with HA tags in TgAa16GL |
| gRNA-3Aa16GL-R | 5′-AACTTGACATCCCCATTTAC |  |
